# Authors' Reply to "Anatomic Twist to a Straightforward Ablation'

**DOI:** 10.1016/s0972-6292(16)30696-9

**Published:** 2013-11-15

**Authors:** Mandeep Singh Randhawa, Harris C Taylor, Robert D Mosteller

**Affiliations:** 1 Clinical Associate, Department of Hospital Medicine, Cleveland Clinic Foundation, Cleveland, Ohio; 2 Director Resident Research, Fairview Hospital - Cleveland Clinic, Clinical Professor of Medicine, Case Western Reserve University, School of Medicine, Cleveland, Ohio; 3 Cardiology/Electrophysiology, Heart and Vascular Institute, Cleveland Clinic, Cleveland, Ohio

**Keywords:** Ablation, Anatomic Twist

We appreciate the thoughtful comments of Dr. Chase, Dr. Devi A and Dr. John regarding our case report [1] of a patient who underwent AV junction ablation via a congenitally abnormal venous system. Our patient had an interrupted inferior vena cava which continued as the azygos vein, in turn draining into the superior vena cava. Ablation was successfully performed with the ablation catheter through a long SRO sheath taking this circuitousroute.

Dr. Chase et al. mention potential alternative approaches to achieving iatrogenic AV block, such as introducing the ablation catheter from an internal jugular vein, or using a left-sided approach via trans-arterial access. The latter is highly effective [2,3], but certainly has potential complications (such as systemic embolization or injury to the aortic valve or coronary arteries), and we would have considered this only if the transvenous approach had failed. A right internal jugular venous approach, on the otherhand, would have been most reasonable and the preferred venous approach (the left subclavian vein had been used for prior pacemaker lead placement) had thepatient's exact anomaly been known to the operators. However, since venous access had been obtained through the femoral vein, an attempt via this approach was thought to be worthwhile. The large caliber of the azygos vein, essentially the same as that of the inferior vena cava in a normal subject, allowed the safe advancement of the ablation catheter into the superior vena cava and the right atrium. The SRO sheath, while possessing some degree of stiffness, was similarly advanced into the right heart, always tracking over the ablation catheter to minimize the small risk of venous injury in the process. The primary operator in our procedure had over two decades of experience in catheter manipulation as well as in coronary arteriography and venography. He would have abandoned the inferior approach had the technical difficulty in advancing the ablation apparatus been excessive. Dr. Chase et al. make an excellent point that a less-experienced operator might be advised to utilize the internal jugular approach instead. We thank them for their insightful comments.
